# Pulmonary Kaposi's sarcoma after heart transplantation: a case report

**DOI:** 10.1186/1752-1947-4-206

**Published:** 2010-07-05

**Authors:** Florian R Fritzsche, Michaela Tutic, Isabelle Opitz, Roger Hunziker, Glen Kristiansen, Matteo Montani

**Affiliations:** 1 Institute of Surgical Pathology, Universit228;tsSpital Zürich, Schmelzbergstr. 12, 8091 Zurich, Switzerland; 2 Devision of Thoracic Surgery, Universit228;tsSpital Zürich, Schmelzbergstr. 12, 8091 Zurich, Switzerland; 3 Institute for Diagnostic Radiology, Universit228;tsSpital Zürich, Schmelzbergstr. 12, 8091 Zurich, Switzerland

## Abstract

**Introduction:**

Kaposi's sarcomas have been associated with different conditions of immunosuppression and are also known to be a typical complication of solid organ transplantations.

**Case presentation:**

We report the case of a 65-year-old Turkish man with a history of heart transplantation 10 months ago who presented for clarification of his dyspnea. The patient had a known history of chronic obstructive pulmonary disease and a smoking history of 40 pack years. Radiologically, three progressively growing intra-pulmonary nodules were detected. The histology was diagnostic for a Kaposi's sarcoma. Visceral and especially primary intra-pulmonary Kaposi's sarcomas are very rare and have been described to have a rather unfavorable prognosis.

**Conclusions:**

Even with a history suggestive for conventional lung cancer, Kaposi's sarcomas should be considered in patients after transplantation of solid organs. It should be noted that in a minority of cases this tumor exists in the absence of the typical cutaneous lesions.

## Introduction

Kaposi's sarcomas are infrequent malignant tumors in the USA derived from vasoformative mesenchyme with a higher regional prevalence (endemic form) in the Mediterranean basin, the Middle East and Eastern Europe and in immunocompromised patients [1]. The latter encompasses mainly patients with acquired immunodeficiency syndrome (AIDS) and to a lesser extent individuals with other causes of compromised immunity, for example after solid organ transplantation (so called iatrogenic forms). It was estimated that the frequency of Kaposi's sarcoma in AIDS patients is still several hundred times higher than that in immunocompromised patients of other causes [1]. Classic (cutaneous) Kaposi's sarcoma typically manifests as bluish-red, well demarcated, painless dermal maculae, plaques or nodules in the distal lower extremities, which can become pedunculated and may ulcerate. Advanced lesions might display a brown color and hyper-keratotic surfaces. In addition to mucosal involvements visceral tumors (lymph nodes, gastrointestinal tract, lung, spleen) have been described for advanced cases and rarely as primary manifestations [1]. Most Kaposi's sarcomas were found in individuals older than 50 years with a clear male predominance. The course of these tumors is generally prolonged, although short courses have been described [1].

In the 1990 s the connection between the human herpes virus 8 (HHV8) and Kaposi's sarcomas was established. Virtually all Kaposi's sarcomas are thought to harbor this virus which can be detected by immunohistochemical and molecular-pathological techniques [1].

In this case report we describe the rare case of a primary intra-pulmonary Kaposi's sarcoma in a human immunodeficiency virus (HIV)-negative 65-year-old man with a history of heart transplantation 10 months previously.

## Case presentation

A 65-year-old Turkish man presented to the hospital for clarification of dyspnea. Computed tomography (CT) showed a small tumor in the lower lobe of the left lung. Subsequent bronchial lavage was non-contributive concerning possible viral, bacterial (including mycobacteria) and fungal causes or malignancy. It was decided to perform a follow-up CT in three months.

**Figure 1:**
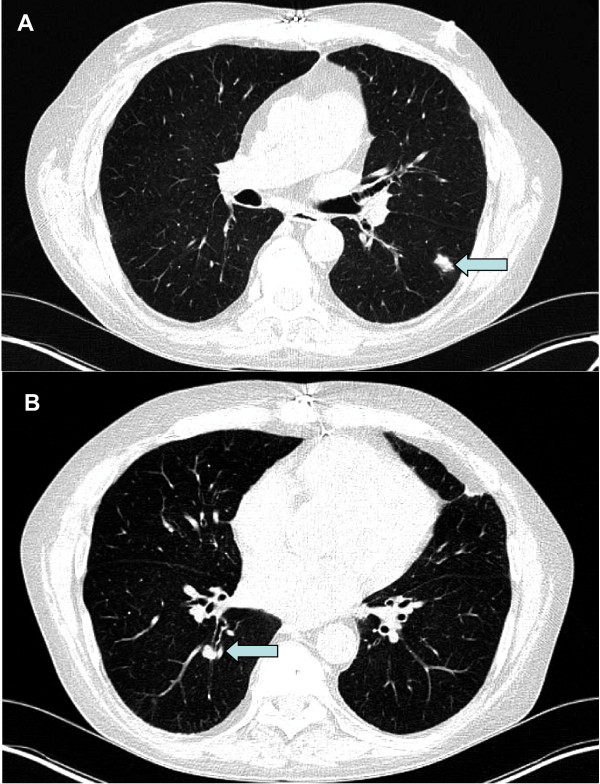
**Lung imaging**. Computed tomography displaying the progressively growing tumors (arrows) in the left (**A**) and right (**B**) lung.

Our patient had a medical history of heart transplantation 10 months previously due to biventricular heart insufficiency, chronic atrial fibrillation and multi-vessel coronary heart disease. Additionally, he had known chronic obstructive pulmonary disease (COPD; smoking history of 40 pack years), hyper-cholesterinemia, substituted hypothyroidism, articular gout, normocytic normochromic anemia, steroid induced myopathia and obesity, hypertrichosis (following therapy with cyclosporine) and a recently polymerase chain reaction (PCR)-confirmed cytomegaly virus infection. Follow-up CT after three and four months displayed three progressively growing tumors in the upper and lower lobe of the left lung (up to 1.6 cm) and in the lower right lobe (Figure 1A, B). Physical examination revealed no relevant findings. There were no palpable lymph nodes, no signs of tumors on the extremities and the skin.

**Figure 2:**
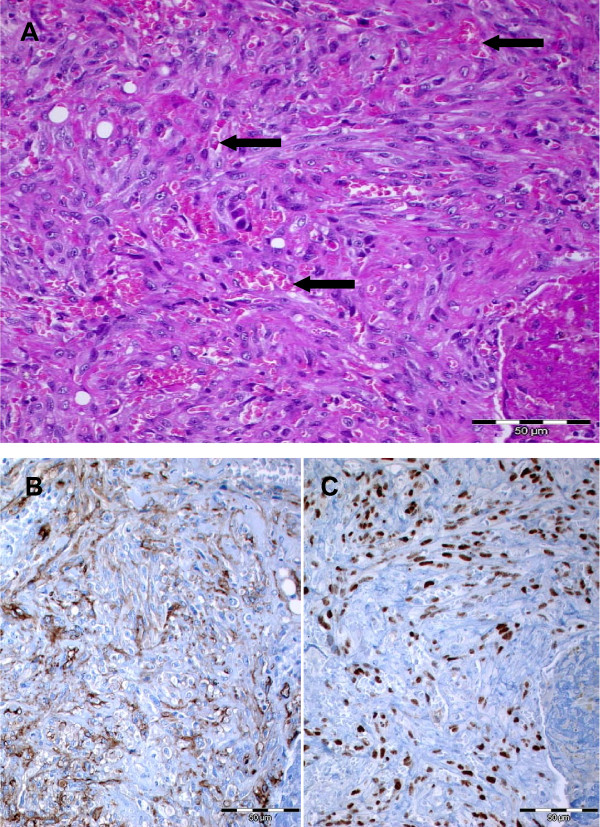
**Histological images**. Hematoxylin and eosin staining (**A**) of the intra-pulmonary tumor with the distinctive slit-like vascular clefts (arrows). The tumor cells are positive for the vascular marker CD31 (**B**) and display a nuclear staining for HHV8 (**C**).

Since the above described lesions were not thoracoscopically detectable, an open thoracotomy was performed. The tumors in the left upper and lower lobe were surgically removed by wedge resection. One of the tumors was sent for intra-operative frozen section. Macroscopically, the tumor was red-brown and well demarcated from the surrounding parenchyma. Histologically, the frozen section revealed an intra-pulmonary spindle-cell tumor. Intra-operatively the dignity of the tumor could not be definitely determined. On paraffin sections, the hematoxylin and eosin staining in all obtained specimens displayed rather monomorphic spindle-cell tumors (Figure 2A) with slit-like vascular clefts, entrapped erythrocytes and evidence of older hemorrhages. Tumor cells were positive for vascular molecular markers (CD 31, Figure 2B), moderately proliferating, and showed a fine granular nuclear positivity for HHV8 (LNA-1, Figure 2C). These findings were diagnostic for Kaposi's sarcoma.

In the post-operative course our patient was transferred to the intensive care unit for two days for short-term intubation owing to pulmonary edema and reduced diuresis. He recovered well and was transferred back to the normal ward. Heart and lung function were normal. Our patient was able to leave hospital on the tenth post-operative day. Immunosuppressive therapy was changed from cyclosporine to everolimus and a follow-up CT in three months was arranged. In the meantime the immunosuppressive therapy might be further adapted to a possible chemotherapy.

## Discussion

Iatrogenic (post-transplantation) Kaposi's sarcomas are most often detected in patients with a previous history of kidney transplantation, which reflects the higher frequency of these transplantations. The rate of post-transplantation Kaposi's sarcoma was estimated at about 0.5% (range 0.06% to 4.1%). The majority of cases, including the one described, have been of patients originating from the Mediterranean, the Middle East area or African regions, known for the endemic form of this tumor entity. For some of the patients with iatrogenic Kaposi's sarcoma the hypothesis is that the tumor cells originated from infected progenitor cells received from the donated organ.

Larger studies have already described Kaposi's sarcomas following heart transplantation [2,3]. In most of these cases, the tumors were localized in the skin but some tumors also demonstrated visceral involvement (lymph nodes, uterine cervix, gum, tonsils, lung and heart). Likewise, very recently Sharif-Kashani *et al. *reported a case of cutaneous and diffuse pulmonary involvement six months after heart transplantation [4]. Indeed the visceral involvement is supposed to be more frequent following heart or liver transplantations in comparison to renal transplantations [2]. The overall frequency of purely visceral Kaposi's sarcomas is thought to be around 10% after solid organ transplantation [2]. However, case reports describing a solitary involvement of the lung following heart transplantation are extremely rare [5]. Table 1 depicts published cases of purely visceral Kaposi's sarcoma following heart transplantations. We also detected six cases of lung involving, non-cutaneous Kaposi's sarcoma after lung transplantation in the literature.

**Table 1:** Purely visceral Kaposi's sarcomas after heart transplantation

Author; year of publication	No. of patients	Localization
Bhoopchand A; 1986 [9]	1	lymph nodes
Lopez-Rubio F; 1994 [10]	1	bone, lungs, gastrointestinal, lymph nodes and liver
Fonesca R; 1998 [11]	1	gastrointestinal and lung
Jones D; 1998 [12]	1	lymph nodes
Collart F; 2004 [13]	1	gums, tonsils, lymph nodes
Wasywich CA; 2006 [5]	1	lung

Wasywich *et al. *reported the case of a 42-year-old man who had developed multiple intra-pulmonary Kaposi's sarcomas 28 days after heart transplantation [5]. As in our case no other tumor nodules were detected. For the patient in Wasywich's case a remission for about two years was achieved by switching from cyclosporine and azathioprine to sirolimus. However, eventually lymph node metastasis occurred [5].

Cases of primary pulmonary Kaposi's sarcoma were mostly associated with AIDS, but have also been described as rare complications of other solid organ transplantations [6]. Like involvement of other visceral organs, most cases represent primary cutaneous Kaposi's sarcomas with additional visceral components [2,4]. Only one case of an immunocompetent young man with pulmonary Kaposi's sarcoma has been described so far [7].

Typical but unspecific radiological features of Kaposi's sarcoma in such patients were nodular or reticular opacities, pleural effusions and hilar/mediastinal lymphadenopathy.

Interestingly, in contrast to our case, most reports in the literature describe primary and secondary pulmonary Kaposi's sarcoma with multiple nodular manifestations. Although this case underlines that, even in the absence of skin manifestations, one should consider Kaposi's sarcoma as a possible cause of an intra-pulmonary tumor, common lung cancers (especially adenocarcinomas) are also important and frequent differential diagnoses in heart transplant patients.

For post-transplantation patients with Kaposi's sarcoma the five-year survival rate was observed at about 68% and these patients had a rather high rate of visceral involvement (47%) [8]. Since many patients - including our own - received cyclosporine, it is thought that there might be causative connection. Several studies suggest that the omission of cyclosporine might lead to tumor remission [5]. Complete remission of Kaposi's sarcomas was described using a combination of immunomodulation (reduced/changed immunosuppression), irradiation and surgery [8]. However, for patients with visceral involvement, as well as for those with previous heart transplantation, the prognosis was worse than for other post-transplantation Kaposi's sarcoma cases [8]. Woodle *et al. *therefore recommended a more aggressive combination therapy in such cases [8].

## Conclusions

In conclusion we report a rare case of Kaposi's sarcoma following heart transplantation with primary intra-pulmonary manifestation without skin lesions. Even with a history suggestive for lung cancer, Kaposi's sarcoma should be considered as a differential diagnosis in patients after transplantation of solid organs. The prognosis of patients with visceral Kaposi's sarcoma seems to be rather serious and requires intensive follow-up and consideration of different therapeutic measurements.

## Competing interests

The authors declare that they have no competing interests.

## Authors' contributions

FRF, GK and MM interpreted and diagnosed the histological results, conceived the report and provided histological images. MT and IO provided clinical data and contact with patient. RH provided radiological images. All authors contributed to, read and approved the final manuscript.

## Consent

Written informed consent was obtained from the patient for publication of this case report and any accompanying images. A copy of the written consent is available for review by the Editor-in-Chief of this journal.
